# Prolonged social media use is not fundamentally problematic in a Hungarian representative study

**DOI:** 10.1038/s41598-026-36896-2

**Published:** 2026-01-28

**Authors:** Ágnes Zsila, Bulcsu Bognár, Reza Shabahang

**Affiliations:** 1 Institute of Psychology, ELTE Eötvös Loránd University, Budapest, Hungary; 2 Department of Sociology and Communication, Faculty of Economic and Social Sciences, Budapest University of Technology and Economics, Műegyetem rkp. 3, H-1111, Budapest, Hungary; 3 Department of Psychology, College of Education, Psychology and Social Work, Flinders University, Adelaide, Australia; 4 Department of Business, College of Business, Government and Law, Flinders University, Adelaide, Australia

**Keywords:** Addiction, Problematic social media use, Representative sample, Social networking sites, Psychology, Human behaviour

## Abstract

Excessive time spent using social media is often considered a core characteristic of technology-related addictions; however, there is growing evidence that prolonged use may be only weakly associated with symptoms of problematic use. Studies on the divergent associations of psychological factors with prolonged and problematic social media use are still scarce, particularly in adult, representative samples. Based on self-report data, this study aims to address this gap by investigating possible divergent associations of prolonged and problematic social media use among active and passive use, and indicators of psychological distress in a nationally representative sample of adults. A hybrid data collection procedure was employed, including offline and online panelists. The sample comprised 807 Hungarian adults (53.41% women, *M*_age_ = 46.61 years, *SD* = 16.58). A structural equation model (SEM) was constructed. Both active and passive use predicted more time spent on social media, while problematic social media use symptom severity was predicted by active use, social comparison, and psychological distress. The explanatory power of these factors was notable in problematic social media use (58%) and modest for time spent on social media (16%). These findings provide further evidence that prolonged and problematic social media use may be different in their qualitative characteristics.

**Supplementary Information:**

The online version contains supplementary material available at 10.1038/s41598-026-36896-2.

## Introduction

Social media can fulfill various needs, including socialization, information-seeking, alleviating boredom, entertainment, escaping real life and everyday problems, or expressing one’s identity (see social media use within the framework of the Uses and Gratifications Theory^1^. Individual needs^2^, motivations^3^, and psychological characteristics^4^ can determine the ways of engagement with social media. Recently, problematic social media use has become a growing concern with the proliferation of social media platforms^5^. Problematic social media use is often characterized by the loss of control over the activity and deterioration in functioning in work or school environments, relationships, and other life aspects as a result of compulsive social media use^6^. Drawing on addiction-related theoretical frameworks—particularly the Components Model of Addiction^7^—problematic use has been conceptualized as comprising six key components (i.e., symptoms): salience (preoccupation with social media), tolerance (the need to spend increasing amounts of time on social media), mood modification (using social media to cope with emotional distress), relapse (unsuccessful attempts to reduce usage), withdrawal (experiencing distress when unable to use social media), and conflict (social media use causing disruption in personal, social, or occupational life).

However, ongoing debates question whether these criteria adequately capture problematic social media use. Some scholars argue that not all components are equally applicable or sufficiently distinguish between problematic and non-problematic users^8,9^. Moreover, similar to other forms of potentially problematic media engagement, the classification of problematic social media use as a formal clinical disorder (i.e., social media addiction) remains controversial and has yet to achieve consensus within the psychiatric and psychological communities^5,10^. Drawing on more recent suggestions included in the Diagnostic and Statistical Manual of Mental Disorders (DSM-5) and the International Classification of Diseases (ICD-11), problematic social media use can be described by some features typical of substance use disorder and behavioral addictions, such as gambling disorder^36^. Specifically, problematic social media use can also be characterized by strong craving or urge to use social media (DSM-5), impaired control (ICD-11), and fear of missing out (a specific feature). It can also involve a preference for online activities over real-life activities to the point of experiencing social, communication, or system-related overload (a specific feature). This problematic use pattern can lead to functional impairment with regard to social, occupational, or recreational activities (DSM-5; ICD-11), and persistent social, interpersonal, or mental health problems due to excessive social media use (DSM-5; ICD-11)^36^.

Beyond the debate on how problematic social media use could be more accurately defined, the overlaps and distinctions between problematic social media use and prolonged social media use have recently attracted increasing research attention. Indeed, there is a need to enhance understanding of problematic social media use, clarifying its convergent and divergent features with other social media use patterns, which can ultimately contribute to a more accurate definition. Previous studies have suggested that prolonged use of social media may not necessarily be problematic^9,11^. However, research on problematic social media use has mostly been conducted among undergraduate and adolescent samples in the past decade, which considerably limits the generalizability of findings on the distinction between high engagement and problematic use^12^. The present study aims to provide further empirical evidence on the possible divergent predictors of prolonged and problematic social media use in a nationally representative sample of Hungarian adults, extending findings on the qualitative differences between these two forms of social media use.

A recent, large-scale study provided a detailed classification of adult social media users, indicating that highly engaged users typically experienced lower levels of psychological distress (i.e., symptoms of depression, anxiety, and stress) compared to problematic users^9^. Studies on problematic online gaming^13^, problematic pornography consumption^14^, and problematic Twitch use^15^ found similar patterns in the association of frequent and problematic use with indicators of psychological distress. Some studies^16,17^ found positive associations between behavioral addictions (e.g., gaming disorder, problematic gambling, and compulsive buying), highlighting the similarities in the underling compulsive and impulsive behavioral mechanisms, neural processes, and personality and mental health correlates across these problematic behaviors. In a similar vein, a recent network analysis showed that while some behavioral addictions are closely related due to severe overlap between activities (e.g., problematic internet use and problematic social media use), the majority of behavioral addictions should be viewed as “different manifestations of the same underlying disorder”^18^ (p. 18). Studies^13–15^ also found only weak or moderate associations between time spent using social media and problematic use patterns. For instance, the contribution of time spent on social media was negligible (12%) in predicting problematic social media use in the study by Schmelzer et al.^19^. Therefore, the commonly held assumption that the excessive amounts of time spent on social media may be a core feature of addiction^20^ seems to be inaccurate in many cases. These findings also highlight the need for further investigation into the qualitative distinctions between different types of social media use.

Previous research has demonstrated that problematic social media use is a pervasive phenomenon^21^, which is present in all societies and age groups studied to date. The present research aimed to use a sample that ensures the generalizability of the findings to a wider population. The limited number of representative investigations indicates that the findings from the Hungarian population under examination are consistent with those observed in other countries^21^. Indeed, Király et al.^22^ reported that 71% of the Hungarian adult population used social media platforms in the past month preceding the data collection. Social media users spent an average of 10 h a week on these platforms. No difference was found between men and women in either prolonged or problematic social media use, while younger age was associated with higher problematic social media use symptom severity. The common use of social media in the Hungarian adult population underlines the importance of investigating possible divergent associations of the dynamics of social media use (i.e., active and passive use), and social and psychological characteristics (i.e., loneliness, social comparison, and psychological distress) with prolonged and problematic use to draw a clearer picture of possible differences between the two engagement forms in the general adult population.

Studies on the psychological correlates of active (e.g., creating content and interacting with other users) and passive (e.g., scrolling and browsing) social media use provided mixed findings. A common hypothesis is that active use is associated with psychological well-being, as it fosters social interaction, support, and positive feelings, while passive use is associated with psychological distress, as it facilitates social comparison and envy^23^. However, over 70% of studies provided evidence rejecting this hypothesis according to a recent review, finding only nonsignificant or very weak associations between mental health indices (e.g., depression, well-being, and positive and negative affect) and the types of use^23^. Kircaburun et al.^4^ found that passing time (i.e., passive use) was the strongest predictor of problematic social media use among motives, although motives reflecting active use, such as socialization and maintaining relationships, were also significant predictors. Therefore, it was hypothesized that active and passive use would predict both prolonged and problematic social media use in the present study, but the association of passive use would be stronger with problematic use than with prolonged use, while the association of active use would be stronger with prolonged use than with problematic use.

Social comparison, defined as a tendency of individuals to use a person as a reference to determine their own position in the society, level of skills, behavior, or performance^24^, has been associated with both prolonged and problematic social media use^25,26^. Likewise, perceived loneliness was associated with both types of engagement with social media^27,28^, although Seabrook et al.^29^ suggested that social media use can also decrease feelings of loneliness as it provides possibilities for social connection and peer support. Rozgonjuk et al.^30^ also found that higher neuroticism was associated with higher tendencies for social comparison, which was related to passive social media use. In other studies, both social comparison and loneliness have been associated with depression and anxiety^31,32^. These symptoms were strongly associated with problematic social media use, but no definitive linear relationship could be observed with regard to the self-reported time spent on social media in a recent systematic review^33^. Therefore, it was hypothesized that social comparison and loneliness would predict both prolonged and problematic social media use in the present study, but this association would be stronger with problematic use than with prolonged use. Moreover, it was expected that psychological distress (i.e., symptoms of depression, anxiety, and stress) would predict only problematic social media use.

Overall, recent studies suggest that prolonged social media use differs from problematic use in qualitative term and may be associated with different psychological outcomes^13,14^. However, there is still a lack of studies clarifying this distinction with regard to social media use. Previous studies largely investigated the psychological correlates of prolonged and problematic social media use separately. Moreover, previous investigations that relied on adolescent and undergraduate convenience samples produced findings with limited generalizability to the general adult population^34^. This study seeks to fill these gaps in the literature by investigating possible divergent associations of prolonged and problematic social media use across use patterns (i.e., active and passive use), social characteristics (i.e., loneliness and social comparison), and psychological distress (i.e., symptoms of depression, anxiety, and stress) in a comprehensive model, using a nationally representative sample of Hungarian adults. A clearer distinction between prolonged but nonproblematic and problematic social media use could contribute to a more nuanced understanding of the differences between the two use patterns, potentially enabling earlier and more accurate recognition of problematic use patterns, which are often associated with psychological harm. Such a distinction may also help prevent the overpathologization of extensive yet healthy involvement in common online activities^35^, such as social media use. Furthermore, it may contribute to ongoing efforts aimed at the clinical characterization and operationalization of social media use disorder^36^.

## Hypotheses

H1a. Active and passive use, social comparison, and loneliness are expected to predict both prolonged and problematic social media use.

H1b. The association of passive use, loneliness, and social comparison is expected to be stronger with problematic use than with prolonged use.

H1c. The association of active use is expected to be stronger with prolonged use than with problematic use.

H2. Psychological distress is expected to predict only problematic use and not prolonged use.

## Methods

### Participants and procedure

Data collection targeted 800 adult respondents, derived from the hybrid data collection panel of a Hungarian marketing research company, employing an address-based sampling (ABS) method. Data collection was conducted using a mixed-method approach, incorporating both personal and online data collection. Individuals with internet access participated as online panelists (85% of the sample), whereas respondents without internet access or those who expressed unwillingness to respond online were classified as offline panelists (15% of the sample). A self-administered questionnaire was completed by online panelists, while offline panelists were approached by representatives of the marketing research company and completed the questionnaire combined with computer-assisted personal interview (CAPI). Valid responses were weighted using a post-stratification procedure to address the discrepancies in the demographic composition of the sample resulting from non-response and varying inclusion probabilities. Using this weight variable, estimations were adjusted to align with the 2011 Census, ensuring that the sample was representative of the Hungarian adult population in terms of gender, age, education level, and settlement type. After addressing oversampling, the final sample consisted of 807 Hungarian adults (46.59% men, 53.41% women, *M*_age_ = 46.61 years, *SD* = 16.58, age range: 18–86 years). Participants provided informed consent and were informed about the study’s purpose prior to the survey completion. The research protocol received approval from the institutional review board of the principal investigators’ university. The present study employed a cross-sectional design.

### Measures

Basic demographics were assessed, including gender (1 = men, 2 = women) and age. Participants were also asked how often they used social media (1 = never, 2 = rarely, 3 = occasionally, 4 = often, 5 = always) and how much time they spent on social media on an average weekday and weekend day (1 = less than half an hour, 2 = half an hour – 1 h, 3 = 1–2 h, 4 = 3–4 h, 5 = 5–6 h, 6 = more than 6 h) based on self-report. To increase the interpretability of the findings in terms of hours, the time spent on social media was linearized by calculating the midpoints of the intervals (0.5 = less than half an hour, 0.75 = half an hour – 1 h, 1.5 = 1–2 h, 3.5 = 3–4 h, 5.5 = 5–6 h, 6 = more than 6 h). Daily use in hours was computed by multiplying weekday use by 5 and weekend use by 2, and dividing the total by 7. To increase comparability and interpretability of the present findings on social media use in a Hungarian adult representative sample, this process was entirely based on the protocol described by Király et al.^22^. Linearization of time intervals has been used in several studies on online activities, including methodological studies (see^13,15,37–39^). For individuals who reported never using social media (*n* = 28), the self-reported time of use was set to 0, and their data on problematic social media use were set as missing to maintain the representativeness of the sample distribution and ensure adequate weighting. Items of the assessment instruments used in this study were translated into Hungarian and then back-translated by two independent translators with significant experience in the cross-cultural adaptation of self-report measures.

*Problematic social media use symptom severity* was assessed using the Hungarian adaptation of the Bergen Social Media Addiction Scale (BSMAS)^6,40^. The unidimensional scale comprised six items (e.g., “I felt an urge to use social media more and more”) rated on a five-point scale (1 = *never*, 5 = *always*). Previous research has supported the factor structure, construct validity, and reliability of the Hungarian version of the BSMAS^40^. Higher scores on the BSMAS indicated more frequently experienced symptoms of problematic social media use (α = 0.89).

*Active and passive social media use* was measured using items adapted for social media use by Li^41^ from the active and passive media use scale, originally developed by Pagani and Mirabello^42^. The active use dimension comprised four items (e.g., “I comment on others’ posts on social media sites”; α = 0.89), while the passive use dimension consisted of three items (e.g., “I read online discussions on social media sites”; α = 0.85). Items were rated on a seven-point scale (1 = *never*, 7 = *constantly/all the time*). High reliability and well-fitting factor structure were reported in the original study, providing initial evidence for construct validity^41^. Active use was associated with interaction empowerment, while passive use was not associated with either intrapersonal or interaction empowerment, providing support for predictive validity^41^. Higher scores on the respective subscales indicated more frequent engagement in active or passive social media use.

*Social comparison* was assessed using the short version of the Iowa–Netherlands Comparison Orientation Measure (INCOM)^43,44^. The short version of the INCOM consisted of two factors: ability (three items, e.g., “I often compare how I am doing socially [e.g., social skills, popularity] with other people”) and opinion (three items, e.g., “I always like to know what others in a similar situation would do”). Items were rated on a five-point scale (1 = *I disagree strongly*, 5 = *I agree strongly*). The two latent factors were highly correlated in the present study (*r* = 0.77, *p* < 0.001); therefore, a general latent factor (α = 0.78) was used to avoid severe multicollinearity, following the recommendation of Schneider and Schupp^44^, who also found a strong interrelation between the factors. Therefore, higher scores reflected higher tendencies for social comparison in terms of ability and opinion. Pikó et al.^45^ recently used the INCOM on a sample of Hungarian adults, confirming its high reliability and validity, as demonstrated by its positive correlations with constructs such as perfectionism, problematic social media use, loneliness, and fear of missing out.

*Loneliness* was assessed using the eight-item short version of the UCLA Loneliness Scale (ULS–8)^46^. Items (e.g., “I lack companionship”; α = 0.80) were rated on a four-point scale (1 = *never*, 4 = *always*), with higher scores reflecting stronger feelings of loneliness. A recent study on Hungarian elderly adults^47^ demonstrated good psychometric properties of the scale in terms of factor structure, reliability, and validity, based on the positive associations of the ULS–8 with maladaptive emotion regulation strategies and the negative associations with mental health and positive refocusing.

*Psychological distress* was assessed using the short version of the Depression, Anxiety, and Stress Scale (DASS–9)^48,49^. Items (e.g., “I was unable to become enthusiastic about anything”) were rated on a four-point scale (0 = *did not apply to me at all*, 3 = *applied to me very much*,* or most of the time*) with reference to the past month. Due to the extremely high latent inter-factor correlations between the dimensions of depression, anxiety, and stress (ranging from *r* = 0.83 to *r* = 0.95, *p* < 0.001), a general factor termed “psychological distress” (α = 0.91) was utilized to prevent multicollinearity, following the recommendation of Yusoff^49^, who also reported strong inter-factor correlations. Therefore, higher scores on this general factor indicated greater psychological distress, reflecting increased symptoms of depression, anxiety, and stress. The DASS–9 has recently been utilized in Hungarian adult samples and demonstrated high reliability and validity based on its positive associations with celebrity worship^50^, and hopelessness and negative expectations for the future^51^.

### Statistical analysis

To handle the slight biases in the sample composition, a four-dimensional matrix weighting (based on gender, age, educational level, and settlement type) was utilized for the total sample (*N*_weighted_ = 807) during the data analysis. Descriptive statistics were calculated using SPSS 21.0, while a structural equation model (SEM) was constructed using Mplus 7.4^52^. Such SEM models enable a simultaneous estimation of the effects of predictor variables on multiple outcome variables while controlling for the direct effect of covariates, such as gender and age.

Active and passive social media use, social comparison, loneliness, and psychological distress were specified as latent predictor variables; gender and age were specified as observed covariates; and self-reported time spent on social media (as an observed variable, measured in hours) and problematic social media use symptom severity (as a latent variable) were specified as outcome variables in the SEM model. Prior to the SEM model, confirmatory factor analysis (CFA) was conducted on all measures (see SM Table 1). A robust maximum likelihood estimator (MLR) was used in all analyses, as it is robust to non-normal data distributions.

In the SEM model, error covariances between the 3rd and 4th items of the active social media use subscale, the 1 st and 2nd items of the INCOM, the 1 st and 2nd items of the BSMAS, and the 4th and 7th items of the ULS-8 were included based on the modification indices. These items were not deleted in order to preserve the conceptual complexity of the constructs, as defined in the original studies, and to maintain comparability with other studies using the same constructs^40,41,44,46,48,49^. The following fit indices were applied^53,54^: the Comparative Fit Index (CFI; ≥ 0.95 for excellent, ≥ 0.90 for adequate), Tucker-Lewis Index (TLI; ≥ 0.95 for excellent, ≥ 0.90 for adequate), the Root-Mean-Square Error of Approximation (RMSEA; ≤ 0.06 for excellent, ≤ 0.08 for adequate) with its 90% confidence interval (CI), and the Standardized Root-Mean-Square Residuals (SRMR; ≤ 0.05 for excellent, ≤ 0.10 for adequate). Full information maximum likelihood (FIML) method was used to handle missing data while ensuring appropriate weighting.

## Results

### Descriptive statistics on social media use in the sample

A considerable proportion of participants reported using social media always (*n* = 250; 31.61%), often (*n* = 259; 32.74%), or occasionally (*n* = 159; 20.10%). A smaller proportion reported using social media only rarely (*n* = 95; 12.01%) or not at all (*n* = 28; 3.54%). The average daily social media use was 1.88 h (*SD* = 1.65).

### Predictors of self-reported time spent on social media and problematic use

**Fig. 1:**
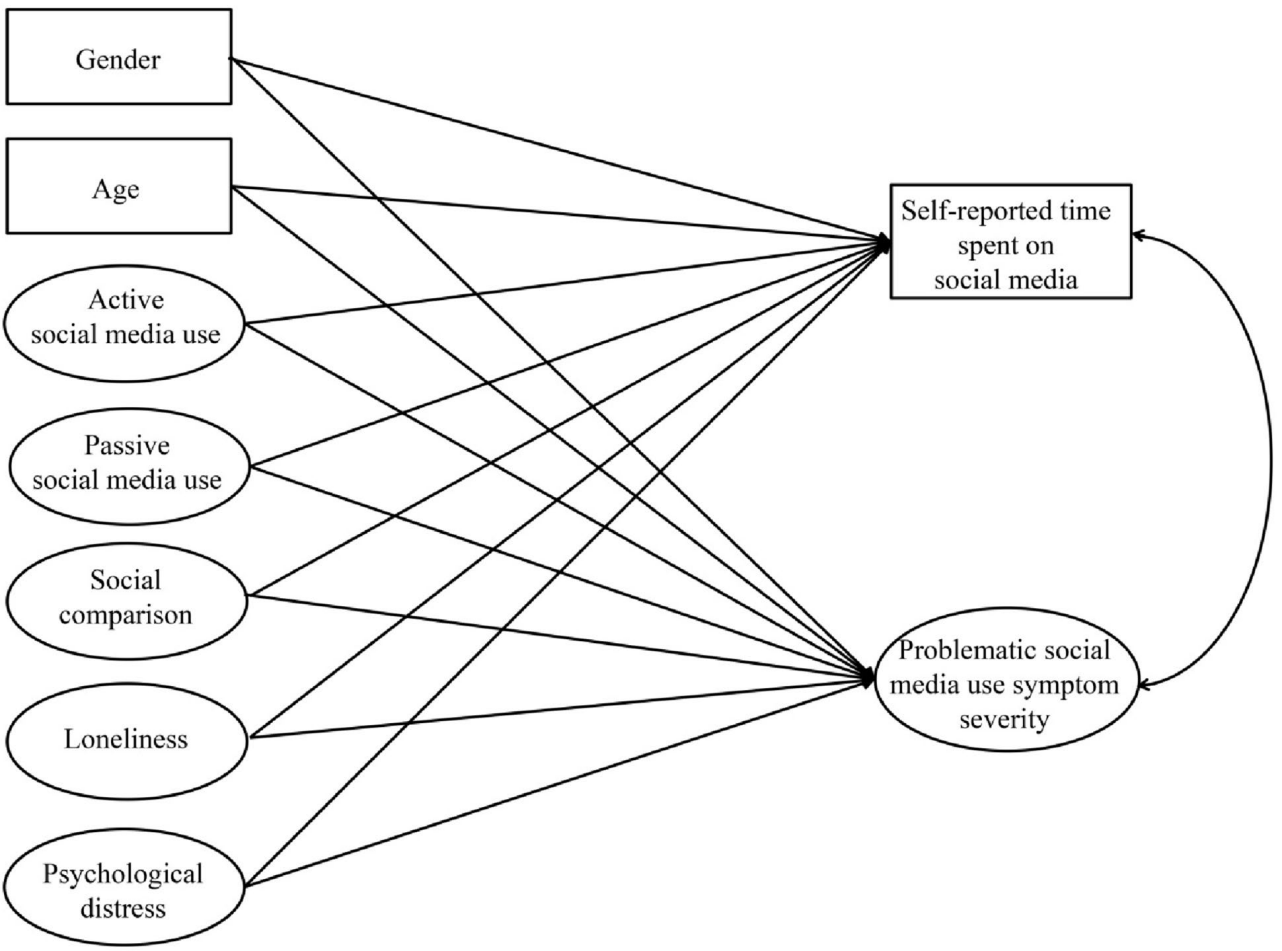
Schematic representation of the structural equation model (SEM) structure. *Notes.* Observed variables are presented in rectangles, while latent variables are presented in ovals. One-headed arrows represent regression paths, while the double-headed arrow represents a correlation between variables.

A SEM model was constructed (*N* = 807) to investigate the convergent and divergent predictors of self-reported time spent on social media and problematic social media use. A schematic representation of the model structure is depicted in Fig. 1, while the results are presented in Table 1. The model fit was adequate (*χ*^*2*^ = 1619.890, *df* = 675, *p* < 0.001; CFI = 0.910, TLI = 0.902, RMSEA = 0.042 [90% CI: 0.039–0.044], SRMR = 0.062).

In addition to younger age, active and passive social media use predicted more time spent on social media. However, these associations were weak, and these predictors explained only a relatively small proportion of the total variance in self-reported time spent on social media (R^2^ = 16%, *p* < 0.001). Younger age and active social media use also predicted more symptoms of problematic social media use. Moreover, higher tendency for social comparison and higher psychological distress were additional significant predictors of problematic social media use symptom severity. The strongest predictors were active social media use and social comparison. Overall, these variables explained a notable proportion of the total variance in problematic social media use symptom severity (R^2^ = 58%, *p* < 0.001). Loneliness was not associated with either type of social media use. Likewise, no association was found between self-reported time spent on social media and problematic social media use in this model (*r* = 0.02, *p* = 0.79).

Overall, although active use predicted both self-reported time spent on social media and problematic social media use, social comparison predicted only problematic social media use, and loneliness was not associated with either prolonged or problematic social media use. Therefore, H1a was only partially supported. Contrary to H1b, passive use was associated only with prolonged use. Social comparison was associated only with problematic use, while loneliness was not associated with either prolonged or problematic social media use. As H1b was formulated based on the expectation that associations proposed in H1a would be present, and these assumptions were not met, H1b was not supported. According to the overlapping CIs in active use across prolonged and problematic social media use, there is no statistically significant difference between the strength of these associations. Therefore, H1c was not supported. As psychological distress predicted only problematic use, H2 was supported.

**Table 1:** Structural equation model (SEM) investigating the predictors of self-reported time spent on social media and problematic social media use symptom severity (*N* = 807).

Predictor variables	Outcome variables					
	Self-reported time spent on social media (hours a day) *R*^2^ = 0.158			Problematic social media use symptom severity *R*^2^ = 0.575		
	β (SE)	95% CI	*p*	β (SE)	95% CI	*p*
Gender	0.06 (0.04)	−0.02; 0.14	0.147	−0.02 (0.04)	−0.09; 0.05	0.567
Age	**−0.16 (0.05)**	−0.25; −0.08	< 0.001	**−0.08 (0.04)**	−0.16; −0.001	0.048
Active social media use	**0.21 (0.08)**	0.05; 0.37	0.010	**0.45 (0.08)**	0.30; 0.60	< 0.001
Passive social media use	**0.18 (0.09)**	0.01; 0.36	0.041	−0.11 (0.08)	−0.27; 0.06	0.198
Social comparison	−0.09 (0.05)	−0.19; 0.01	0.066	**0.42 (0.06)**	0.21; 0.53	< 0.001
Loneliness	0.10 (0.06)	−0.01; 0.22	0.079	0.06 (0.06)	−0.06; 0.18	0.323
Psychological distress	0.01 (0.06)	−0.12; 0.16	0.893	**0.19 (0.06)**	0.07; 0.30	0.002

*Notes*. CI = confidence interval. Boldfaced values are significant at least at *p* < 0.05. Gender, age, and self-reported time spent on social media were observed variables, while all other variables were latent variables in the model.

## Discussion

Several studies have investigated the psychological correlates of social media engagement and problematic use separately^26–28^; however, research on possible divergent predictors remains scarce. This study provided further evidence that prolonged and problematic social media use may be qualitatively different forms of user behavior, with largely divergent associations in terms of active and passive use patterns and mental health indicators. These findings suggest that high levels of social media engagement (i.e., prolonged social media use) are not fundamentally associated with psychological correlates that are closely linked to problematic social media use, which may be adverse to mental health.

The proportion of social media users in the present sample was much higher (96.5%) compared to the prior report by Király et al.^22^, which reported rates of 71% and 75%. There was a notable increase in social media use among the adult population over the five-year interval between the two representative-sample investigations, along with a simultaneous rise in the prevalence of problematic use.

No association was found between self-reported time spent on social media and symptoms of problematic use of social media in the present model. This finding is supported by previous studies indicating only weak or weak-to-moderate associations between self-reported time of use and problematic use symptom severity in relation to video gaming^13^, internet use^37^, and social media use^55^. Similarly, Schmelzer et al^19^. found that the time spent using social media had only a negligible explanatory power for problematic social media use. These findings suggest that prolonged use of social media may not be a reliable predictor of social media addiction risk.

More time spent on social media was predicted only by active and passive use, while none of the psychological characteristics measured in this study were associated with self-reported time of use. Use patterns explained only 16% of the total variance in self-reported time spent on social media. Active use also predicted symptoms of problematic use, which is in line with the findings by Kircaburun et al^4^., indicating that the motives of seeking and maintaining social relationships on social media platforms can also explain problematic social media use symptom severity. A recent meta-analysis further found that users engaging in active social media use tend to report higher well-being and positive emotions, but also more symptoms of anxiety^56^. Moreover, the present results align with previous findings indicating a positive association between active social media use and depressed mood^57^.

By contrast, passive use did not predict problematic social media use in the present study, aligning with some previous studies reporting no direct association between passive social media use and depression^58^ and negative affect^59^. These findings somewhat contradict the common hypothesis that active social media use is mostly associated with positive outcomes, while passive use is associated with adverse mental health outcomes^23^, and instead provide support for the Social Compensation Hypothesis^60^. This hypothesis proposes that users with limited real-life social connections or skills may use social media in an attempt to compensate for these deficiencies through online social networking. This framework may also explain the nonsignificant association between perceived loneliness and prolonged or problematic social media use, as well as the predictive role of social comparison in problematic social media use symptom severity observed in the present study.

Overall, H1a proposing that active and passive use, social comparison, and loneliness would predict both prolonged and problematic use, received only partial support, as both prolonged and problematic use was predicted solely by active use. The strength of association across prolonged and problematic use with active use did not differ, contradicting H1c. Moreover, social comparison predicted only problematic use, while loneliness was not associated with either prolonged or problematic use. Therefore, H1a was only partially supported. The strongest predictors of problematic social media use symptom severity were active use and social comparison. As H1b was constructed based on the assumption that these associations would be confirmed and the strength of associations would differ across prolonged and problematic social media use, H1b was not supported.

Supporting H2, psychological distress (i.e., symptoms of depression, anxiety, and stress) predicted only problematic social media use but not prolonged use, which is consistent with previous findings on this association^61–63^, and aligns with results reported by Peng and Liao^9^, who found that highly engaged social media users experienced fewer symptoms of depression, anxiety, and stress than problematic users.

Overall, social media use patterns and psychological correlates explained a substantial proportion of variance in problematic social media use (58%), whereas their contribution to explaining prolonged social media use was modest (16%). These findings further underline the qualitative discrepancy between prolonged and problematic social media use, consistent with previous research^13–15^.

### Limitations

This study has several limitations that should be addressed in future research. First, due to the cross-sectional study design, causal relationships between psychological variables and social media use patterns cannot be established. Second, although the core variables included in the SEM model were selected based on prior literature, the number of variables may still be limited. Future studies should therefore incorporate additional social and psychological constructs (e.g., fear of missing out, phubbing, or sleep quality) to obtain a more comprehensive understanding of the divergent associations across prolonged and problematic use.

Another important limitation concerns the self-report nature of the measures. Recent studies have demonstrated discrepancies between self-reported and objective measures of social media use time in relation to problematic social media use and psychological correlates (e.g., fear of missing out^64,65^), with objective measures showing weaker or nonsignificant associations. Accordingly, future research should incorporate objective measures (e.g., digitally recorded screen time) to better capture discrepancies between predictors of self-reported versus actual social media use time and problematic use.

Furthermore, general social media use was assessed in the present study, whereas recent evidence suggests that user engagement patterns and associated risks may differ across social media platforms^66,67^. A platform-specific approach in future research could therefore extend knowledge on the role of platform in these associations. Moreover, the BSMAS is not suitable for estimating addiction prevalence rates, as it was not designed for diagnostic purposes and does not fully reflect the most recent diagnostic criteria (e.g., DSM-5; ICD-11). Finally, cross-cultural research is needed to enhance the generalizability of the present findings to other cultural contexts and age groups (e.g., adolescents)^68^. As emphasized by Király et al.^22^, findings from representative samples of Hungarian adults aged 18–64 cannot be generalized to other populations due to differences in age range, methodology (e.g., offline or online data collection), definition of problematic social media use, and operationalization of the measured constructs across studies. Therefore, the results should be interpreted within the context of the present sample^22^.

### Implications and future directions

Recent studies^13–15,35,69^ have highlighted that high engagement in various online activities (e.g., gaming, pornography use) is not necessarily associated with functional impairments often observed in problematic use (e.g., decline in personal performance, deteriorated social relationships, and poorer mental health). For instance, Bőthe et al.^69^ found that sexual functioning problems were negatively associated with frequent pornography use but positively with problematic use. In the context of video gaming, Billieux et al.^35^ emphasized that a clearer distinction between high involvement and problematic involvement can facilitate more accurate screening and diagnosis of gaming disorder. Drawing a clearer line between the two use patterns may also help prevent the overpathologization of frequent yet healthy engagement in an online activity^35^.

In this context, the present findings on the divergent predictors of prolonged and problematic social media use may advance understanding of differences between the two use qualities, contributing to a more nuanced picture of their boundaries. To date, few studies have investigated these differences with regard to social media, using large-scale representative samples, despite the widespread use of social media globally and among Hungarian adults^22^. Indeed, 63.9% of the global population were identified as social media users in February 2025^70^.

The present findings may also inform ongoing efforts toward the classification and clinical characterization of social media use disorder^36^ by providing empirical evidence regarding the divergent and convergent predictors of prolonged and problematic social media use, thereby offering a deeper insight into the different associations of the two use patterns with maladaptive mental states (e.g., psychological distress). This differentiation may support earlier and more accurate recognition of problematic engagement and guide prevention and intervention strategies by focusing on associated mental states that are more indicative of problematic engagement than high yet healthy engagement. Future research should explore further divergent and convergent associations with prolonged and problematic use across a broader range of sociodemographic (e.g., education, socioeconomic status) and psychological factors (e.g., fear of missing out, personality traits) to refine distinctions between problematic and nonproblematic social media engagement.

## Conclusion

Growing evidence suggests that prolonged and problematic engagement in internet-based activities (e.g., video gaming, streaming, pornography viewing) are qualitatively distinct constructs with potentially different psychological consequences^13–15^. However, evidence supporting this distinction in the context of social media use remains limited, particularly in representative samples. Given that many convenience samples (e.g., student samples) may differ substantially from adult populations in personality and attitudinal characteristics^34^, representative samples can provide more valid and generalizable insights.

The present study provided further empirical evidence for the divergent associations of social media use patterns (i.e., active and passive), social characteristics, and psychological distress with prolonged and problematic social media use. While prolonged time spent on social media was associated with active and passive use patterns, problematic use symptoms were predicted by active use, higher social comparison tendencies, and psychological distress, which together explained a substantial proportion of variance in problematic use symptom severity (58%), compared to self-reported time spent on social media (16%). These findings underscore the importance of distinguishing prolonged social media use from problematic use in prevention and intervention efforts. Targeting social comparison and evaluating the content of active use (e.g., quality of social relationships on social media) could also contribute to the efficacy of such programs. For instance, a recent study by Shabahang et al^71^. has suggested that mindful use of social media can possibly decrease psychological distress. Overall, the results highlight the need for targeted, psychologically informed approaches to reduce problematic social media use without pathologizing high but nonproblematic engagement.

## Supplementary Information

Below is the link to the electronic supplementary material.

Supplementary Material 1

## Data Availability

Data is available upon request by email to the corresponding author.
