# Supplementary material for: Prolonged social media use is not fundamentally problematic in a Hungarian representative study
Source: Sci Rep. 2026 Jan 28;16:6488. doi: 10.1038/s41598-026-36896-2 (PMC12909968; doi:10.1038/s41598-026-36896-2)
Supplement: Supplementary file 1 — Supplementary Material 1 [file 41598_2026_36896_MOESM1_ESM.docx]

**Supplementary materials**

**SM Table 1**

*Confirmatory factor analysis (CFA) for the measures included in the structural equation model (SEM; N = 807)*

| **Measure** | ***χ^2^(df)*** | **CFI** | **TLI** | ***RMSEA (90% CI)*** | ***SRMR*** |
| --- | --- | --- | --- | --- | --- |
| *BSMAS* | 28.316 (8)*** | 0.981 | 0.964 | 0.057 (0.035–0.081) | 0.023 |
| *Active and passive social media use* | 30.416 (12)** | 0.986 | 0.975 | 0.044 (0.025–0.063) | 0.026 |
| *INCOM* | 33.955 (8)*** | 0.970 | 0.944 | 0.063 (0.042–0.086) | 0.044 |
| *ULS-8* | 62.933 (19)*** | 0.970 | 0.956 | 0.054 (0.039–0.069) | 0.032 |
| *DASS-9* | 164.821 (27)*** | 0.928 | 0.904 | 0.080 (0.068–0.091) | 0.043 |

*Notes. ***p* < 0.001, ***p* < 0.01. CFI = Comparative Fit Index; TLI = Tucker-Lewis Index; RMSEA = Root-Mean-Square Error of Approximation; SRMR = Standardized Root-Mean-Square Residuals; CI = confidence interval; BSMAS = Bergen Social Media Addiction Scale; INCOM = Iowa–Netherlands Comparison Orientation Measure; ULS-8 = UCLA Loneliness Scale; DASS-9 = Depression, Anxiety, and Stress Scale. Error covariances were defined between the 1^st^ and 2^nd^ item of the BSMAS, the 3^rd^ and 4^th^ item of the active social media use subscale, the 1^st^ and 2^nd^ item of the INCOM, and the 4^th^ and 7^th^ item of the ULS-8 according to the modification indices.
